# The Integration of Gold Nanoparticles into Dental Biomaterials as a Novel Approach for Clinical Advancement: A Narrative Review

**DOI:** 10.3390/jfb15100291

**Published:** 2024-09-30

**Authors:** Saharat Jongrungsomran, Dakrong Pissuwan, Apichai Yavirach, Chaiy Rungsiyakull, Pimduen Rungsiyakull

**Affiliations:** 1 Department of Prosthodontics, Faculty of Dentistry, Chiang Mai University, Chiang Mai 50200, Thailand; saharat_j@cmu.ac.th (S.J.); apichai.y@cmu.ac.th (A.Y.); 2 Nanobiotechnology and Nanobiomaterials Research Laboratory, School of Materials Science and Innovation, Faculty of Science, Mahidol University, Bangkok 10400, Thailand; dakrong.pis@mahidol.ac.th; 3 Department of Mechanical Engineering, Faculty of Engineering, Chiang Mai University, Chiang Mai 50200, Thailand; chaiy.rungsiyakull@cmu.ac.th

**Keywords:** gold nanoparticles, dental biomaterial, antimicrobial activity, osteogenic activity, cell adhesion, cell proliferation, cell differentiation

## Abstract

Gold nanoparticles (AuNPs) have gained significant attention in the biomedical field owing to their versatile properties. AuNPs can be customized by modifying their size, shape and surface characteristics. In recent years, extensive research has explored the integration of AuNPs into various dental materials, including titanium, polymethylmethacrylate (PMMA) and resin composites. This review aims to summarize the advancements in the application of modified AuNPs in dental materials and to assess their effects on related cellular processes in the dental field. Relevant articles published in English on AuNPs in association with dental materials were identified through a systematic search of the PubMed/MEDLINE, Embase, Scopus and ScienceDirect databases from January 2014 to April 2024. Future prospects for the utilization of AuNPs in the field of dentistry are surveyed.

## 1. Introduction

Nanotechnology has greatly enhanced modern healthcare, resulting in improved patient well-being and quality of life. It has facilitated the advancement of novel and more efficient pharmaceuticals, medical procedures and diagnostic instruments. Nanotechnology has numerous applications in dentistry and is crucial to the development of advanced dental equipment used in treatment. Nanoparticles (NPs) can be precisely manipulated because of their size, which is at the nanoscale level (less than 100 nm), as well as their morphology. The interaction of NPs at the molecular level is more efficient than that of micro- or macro-sized particles. NPs can be easily controlled and used in a wide range of applications because of their large surface area. The prerequisites for utilizing NPs include being biologically inactive, not causing cancer, possessing adequate mechanical durability and being resilient to the internal conditions of the body. There are several widely recognized types of nanoparticles, including carbon-based nanoparticles, metal nanoparticles, ceramic nanoparticles, polymeric nanoparticles and lipid-based nanoparticles; these can be distinguished based on their features. In recent years, numerous researchers have highlighted the significant influence of NPs in the fields of tissue engineering and regenerative medicine [1,2].

AuNPs are commonly used in the medical field, in a variety of applications. They have facilitated the development of effective drugs, medical techniques and diagnostic tools. AuNPs can be controlled at the nanoscale level, including their shape and structure, and have recently been reported to show favorable antimicrobial performance, anticancer effects and antioxidant and anti-inflammatory activities with less toxicity than other metal NPs. Their relative biocompatibility and easy synthesis make them suitable for various biomedical applications, such as drug delivery, imaging and therapy. The factors that affect the properties are the size, structure and surface modifications [1,2]. Moreover, many studies have shown that AuNPs can promote stem cell differentiation, especially in mesenchymal stem cells (MSCs) [3,4,5,6,7,8]. The surface of AuNPs can be stabilized by using various molecules [9,10,11]. Various studies have shown that AuNPs are useful in biomedical applications, including target cell therapy, tissue engineering and regenerative medicine [1,2,8,12,13,14]. Consequently, AuNPs have been engineered in various forms to enhance their efficacy in these applications.

Firstly, the size of AuNPs is dominantly dependent on the cellular uptake and biological distribution [15,16,17]. The uptake and penetration of smaller AuNPs in targeted cells are influenced by their size, which makes them more prone to aggregation in the target cells [18]. Smaller AuNPs usually exhibit higher catalytic efficiency due to their increased surface area-to-volume ratio, providing more active sites for catalytic reactions [19]. The antimicrobial properties of nanoparticles, which can be attributed to their extremely small size, which is up to 250 times smaller than that of bacteria, result in a higher cellular uptake and excess reactive oxygen species (ROS). However, it is important to note that 4 nm spherical AuNPs have a greater tendency to cause cytotoxicity and unfavorable adipogenic differentiation instead of osteogenic differentiation compared to 40 nm AuNPs [8]. AuNPs larger than 80 nm may have difficulty penetrating the cell membrane, as with smaller AuNPs, but they can adhere to bacterial cells to increase tension in the membrane and lead to cell rupture [20]. Secondly, AuNPs adopt a variety of shapes, each with unique properties and applications. In addition to their size, the rate at which AuNPs are taken up by cells is also influenced by their shape, which ultimately affects the number of AuNPs that accumulate inside cells [8,16]. Numerous shapes of AuNPs can be produced, but the most prevalent are spherical and rod-shaped, which are relatively straightforward to synthesize. According to recent findings, spherical AuNPs have demonstrated a greater ability to enhance osteogenic activity and calcium deposition in human MSCs (hMSCs) compared to rod-shaped AuNPs [8]. Another type, star-shaped AuNPs, have been employed in surface-enhanced Raman scattering (SERS), photothermal therapy (PTT) and biological imaging applications. AuNPs can act as either the core or shell component in core–shell structures, resulting in increased stability, high biocompatibility and the ability to be functionalized for drug delivery systems [21]. Lastly, surface modifications play an important role in the application of the target cell [16]. The surface alteration not only improves cell activities but also stabilizes nanoparticles, allowing them to diffuse in bodily fluids [8]. AuNPs coated with specific molecules can attach to specific receptors, resulting in active targeting. Furthermore, passive targeting is dependent on the efficiency of cellular absorption [16]. 

Biomaterials have been used in various applications in dentistry. These materials exhibit promising mechanical properties and biocompatibility in the oral cavity. However, there remains the potential to enhance their performance and longevity. Previous investigations revealed alterations made to dental materials such as titanium [22], PMMA [23] and resin composites by the incorporation of AuNPs [24]. The effective combination of AuNPs with zirconia or polyetheretherketone (PEEK) has also been demonstrated [25]. Titanium has numerous applications in dentistry, exhibiting excellent success rates and eliciting positive biological reactions upon contact with living tissues. Titanium is commonly used in dental implants and its components [26]. Polymeric materials such as PMMA and resin composites have been developed for use in temporary fixed or removable dental prostheses [20,27]. Resin composites have also emerged as materials for direct restoration and modern luting cements [28,29]. Furthermore, nanotechnology modifications such as coatings, sputtering or even mixing on dental materials have been investigated for improving mechanical and biological properties and allowing for biomedical applications [1,2]. These biomaterials are essential to many dental operations and offer a range of options for clinicians to choose from based on the specific requirements and desired outcomes.

As previously noted, AuNPs have proven to be highly effective in a range of biomedical applications, including drug delivery systems [30], enhancing the effectiveness of antimicrobial and anticancer drugs [31,32], photothermal cancer therapy [33] and diagnostic assays [34,35]. Although numerous studies have investigated the use of AuNPs in other fields of biomedicine, there is a lack of research on the long-term biocompatibility and mechanical stability of AuNPs when incorporated into dental materials. In addition, the impact of surface changes on the clinical results of these materials has not been extensively investigated. Given their success in these areas, it is an attractive prospect to investigate their potential use in dental applications. Therefore, this article aims to summarize the properties of AuNPs with various modifications, when used for dental materials, and review their effects on related cells in the field of dentistry.

## 2. Methodology

**Figure 1:**
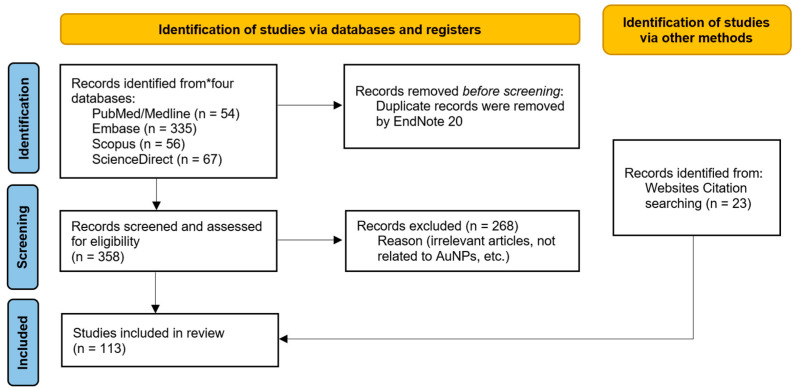
Flow diagram of search procedure.

An electronic search was performed by using the PubMed/Medline, Embase, Scopus and ScienceDirect databases with the following combinations of keywords: (“Gold nanoparticles” OR (“Gold” AND “Nanoparticle”) AND (“Dental” OR “Dentistry” OR “Dental applications”). The search was limited to English-language articles published from January 2014 to April 2024. The electronic results included 512 articles; we eliminated duplicate articles via software for managing references (EndNote 20; Thomson Reuters, Philadelphia, PA, USA) for a total of 358 articles. Following the initial search, the titles and abstracts were reviewed to find relevant papers. Studies that were not conducted in the dentistry field and did not involve the use of AuNPs were excluded. Additionally, articles obtained by electronic hand-searching were included. Finally, we chose 113 articles to include in this comprehensive review (Figure 1). The search terms are shown in Table 1.

## 3. Results

### 3.1. Overview of AuNPs in the Biomedical Field

**Figure 2:**
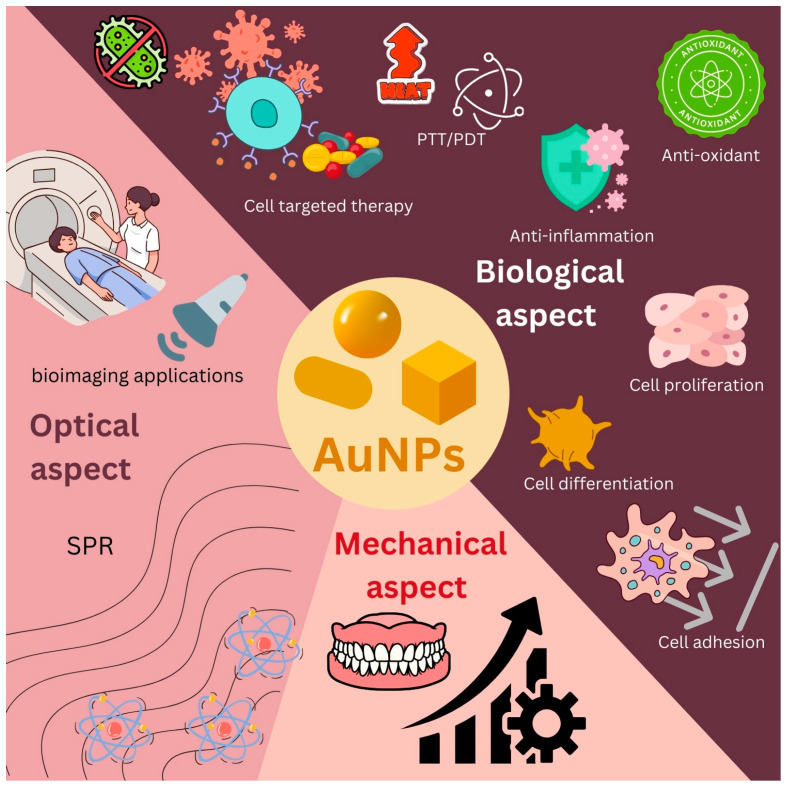
AuNPs in biomedical fields.

AuNPs have been extensively utilized in various biomedical fields due to their unique properties and versatility. They are employed in cancer treatment, biomedical imaging, drug delivery systems, for the improvement of biomaterial properties and in other applications. Through biofunctionalization, AuNPs can be adapted for use in drug administration, vaccine development, sensing and imaging applications, demonstrating considerable potential in these domains. Furthermore, AuNPs play a critical role in tissue engineering and regenerative medicine, offering benefits such as tissue development and precise drug delivery. They can also be created with various forms and dimensions, which enhances their characteristics and potential uses [36]. AuNPs are plasmonic nanoparticles that appear to be less toxic. Because AuNPs are inert, stable and biocompatible, they have been frequently studied for their potential use in biosensing and drug delivery applications. The use of chemical molecules with antibacterial properties to coat AuNPs can offer significant advantages in terms of harnessing their benefits. Consequently, they are promising candidates for delivering targeted antibacterial actions [12]. The size, structure and surface modification of AuNPs have an impact on their characteristics. Furthermore, numerous studies have demonstrated that AuNPs, due to their ultra-small size, large surface-area-to-mass ratio and increased chemical reactivity, are useful in the biomedical field [1,2,8]. Research on AuNPs spans various biomedical fields including dental applications, which can be broadly categorized into three major areas: biological, optical and mechanical (Figure 2). A summary of the studies focusing on the use of AuNPs in biomedical fields is given in Table 2.

**Table 2:** Properties of AuNP-based nanomaterials in biomedical fields.

Property	AuNP Molecule			Associated Cell or Biomarker Assay	Finding	Reference
	Size (nm)	Shape	Surface Modification			
Antimicrobial and antiplaque	2	Nanoclusters	Mesoporous silica nanoparticle-lysozyme-functionalized gold nanoclusters with kanamycin	*E. coli*	A mixed-matrix membrane coating controllably released an antimicrobial agent to instantly inhibit bacterial growth that was only triggered by bacteria.	[37]
	N/A	Spherical with core–shell structure	Core–shell magnetic nanoparticles	*S. mutans*, *E. coli* and *C. albicans*	MNP@Au decreased the adhesion of Gram-positive bacteria cells and fungal cells by 65% and Gram-negative bacteria cells by 45% without influencing the rheological and physicochemical properties of artificial saliva.	[38]
	AuNPs/CS = 16.21 nm AuNPs/CS-Cur = 20.89 nm	Spherical	Chitosan (AuNPs/CS) Chitosan–curcumin (AuNPs/CS-Cur)	*S. mutans* and *E. coli*	AuNP/CS-Cur nanocomposites released curcumin in a pH-dependent manner, which may facilitate the drug to be delivered to the acidic bacterial infection environment. AuNP/CS-Cur possessed the characteristics of electrostatic targeting and photodynamic and photothermal antibacterial therapy; this would become an efficient and safe antibacterial nano-platform and provide new ideas for the treatment of bacterial infection.	[39]
	5–15	Spherical	None	*S. mutans* and *E. coli*	Green synthesized AuNPs exhibited significant antibacterial activity against *S. mutans* and *E. coli*, the same as the commercial ampicillin, in a dose-dependent trend.	[40]
	45–70	Spherical	None	*S. aureus* and *S. mutans*	Green synthesized AuNPs from wheat bran exhibited significant antibacterial activity against *S. aureus* and *S. mutans.*	[41]
	18–35	Spherical	None	*C. albicans*, *S. mutans*, *S. aureus* and *E. coli*	The antimicrobial property for AuNPs was assessed using a zone of inhibition test. They found that AuNPs exhibited excellent antimicrobial activity on *C. albicans* and intermediate antimicrobial potential on *S. mutans*, *S. aureus* and *E. coli.*	[42]
	25, 60, 90	N/A	None	*S. mutans*, *S. sanguinis* and *S. salivarius.*	The smallest size mediated the fast bactericidal activity and showed the most potent antibacterial activity. The 25 nm AuNP was more potent than chlorhexidine against evaluated standard species of *S.mutans*, *S.salivarius* and *S.sanguinis*.	[43]
	43	Spherical	None	*S. oralis*	Au NPs at 100 ppm and 50 ppm concentrations were equally as effective as the CHX against *S. oralis*.	[44]
	20	Spherical	Curcumin	*S. aureus*, *E. faecalis* and *C. albicans*	The antibacterial action of CuAUNPs was seen to be powerful against *S. aureus*, *E. faecalis* and *C. albicans* at a concentration of 100 µg/mL.	[45]
	50	Cube	None	*Candida species; C. albicans*, *C. glabrata* and *C. tropicalis*	The antifungal properties of gold nanocubes against *Candida species* isolates were greater than gold nanospheres and wires.	[46]
	32–41	Nanocages	Antibiotic-loaded, antibody-conjugated, polydopamine (PDA); Staphylococcal protein A (Spa) and an antibiotic (daptomycin, ceftaroline, gentamicin and vancomycin)	*S. aureus*, methicillin-resistant *S. aureus* (MRSA) and *P. aeruginosa*	Daptomycin-loaded AuNCs that are linked to antibodies targeting two distinct lipoproteins of *S. aureus* successfully eradicate MRSA within a biofilm setting. The effectiveness of ceftaroline and vancomycin-loaded AuNCs, which were linked to anti-Spa antibodies, was observed to be lower compared to daptomycin-loaded AuNCs that were also linked to the same antibody. On the other hand, AuNCs loaded with gentamicin and linked to an antibody that targets a specific outer membrane protein were extremely successful in combating *P. aeruginosa* biofilms. The effectiveness of antibiotic-loaded, antibody-conjugated, PDA AuNCs relies on the capacity to induce both a fatal photothermal effect and the controlled release of a significant amount of antibiotic through heat.	[47]
	30	Colloidal	None	*S. mutans*	The combination application of low-temperature plasma and AuNPs resulted in substantial cellular rupture, leading to the release of intracellular constituents from several cells, while AuNPs alone did not showed any bactericidal effect.	[48]
	8–12	Nanoclusters	Methylene blue	*S. mutans*	Bovine serum albumin-capped gold nanoclusters conjugating with methylene blue demonstrated significant antibacterial effects against *S. mutans* when subjected to white-light LED irradiation for approximately 1 min.	[49]
Anti-inflammation and antioxidant	45–70	Spherical	None	DPPH assay	Green synthesized AuNPs from wheat bran possessed high antioxidant activity, which augmented in a dose-dependent manner.	[41]
	20	Spherical	Curcumin	DPPH assay and albumin denaturation assay	At a concentration of 50 μg/mL, CuAuNp demonstrated a maximum scavenging performance of 90.3% against DPPH. CuAuNP shown 79.6% more anti-inflammatory activity at a concentration of 50 μg/mL compared to the conventional medication diclofenac.	[45]
	2–35	Spherical	None	Protein (bovine serum albumin; BSA) denaturation inhibitory DPPH assay	Biosynthesized red sandal AuNP showed 83% (the highest) inhibitory activity of DPPH radicals at the highest concentration of 50 μg/mL. The highest inhibition and maximum protective activity of red sandal AuNP was 80.5% at a concentration of 50 μg/mL. AuNPs exhibited good antioxidant and anti-inflammatory properties.	[50]
	30	Spherical	Ursodeoxycholic acid (UDCA)	Nitric oxide (NO) test and enzyme-linked immunosorbent assay (ELISA)	The thermal effects of NIR-irradiated AuNP-UDCA inhibited the production of inflammatory cytokines from activated macrophages in vitro. The anti-inflammatory effects produced by GNP-UDCA under NIR irradiation were also shown in in vivo conditions using spinal-cord-injured rats.	[51]
	70	Spherical	None	DPPH assay	Biosynthesized AuNPs showed improved antioxidative action at a 1000 μg/mL concentration.	[52]
Anticancer activity	N/A	Spherical	*Siberian ginseng* aqueous extract	Rhodamine 123, H2DCFDA and dual AO/EtBr staining techniques	SG-AuNPs increased the reactive oxygen species and decreased the mitochondrial membrane potential, which induced apoptosis in melanoma cells, and it possessed an anticancer property.	[53]
	50	Spherical	None	Human breast cancer cell lines (MCF-7)	The most effective MCF-7 inhibition was seen when the concentration of AuNPs was 50 μg/mL and they were incubated for 4 h prior to irradiation with the lowest laser power of 0.002 W at a dosage of 0.61 J/cm^2^ for 60 s.	[54]
Cell differentiation	29–38	Spherical	mPEG-SH, the cell-penetrating peptide RGD (RGDRGDRGDRGDPGC) and the mitochondria localization signal (MLALLGWWWFFSRKKC) MLS@RGD-PEG	DPSCs ALP assay kit Ca^2+^ assay kit ATP assay kit	Mitochondrial nanoprobes (MT-AuNPs/MLS@RGD-PEG-AuNPs) showed the potential molecular mechanisms of the accelerated differentiation of DPSCs, where the MMP was increased to induce more ATP production through the thermoplasmonic effect of AuNPs by in situ SERS, the results of which demonstrated that the expression of glucose and hydroxyproline increased within DPSCs during the cell differentiation process.	[55]
	18	Spherical	None	ALP, qRT-PCR of COLIα, RUNX2 and OCN	The addition of AuNPs boosted the behavior of hDPSCs on CPC. This improvement was observed in terms of increased cell adhesion (about double the amount of cell spreading) and proliferation, as well as enhanced osteogenic differentiation (approximately two to three times higher after 14 days).	[56]
Osteoinductive activity	72–88	Spherical	None	MTT (3-(4, 5-dimethylthiazol-2-yl)-2,5-diphenyltetrazolium bromide) assay	AuNPs produced showed exceptional stability in various blood components. Furthermore, the AuNPs were determined to be non-toxic when assessed for their compatibility with periodontal fibroblasts and erythrocytes, indicating their suitability for use in biomedical applications. When GNPs were exposed to MG-63 cell lines, there was a higher percentage of viable cells compared to the control group, indicating that AuNPs could induce bone formation.	[57]
	28–32	N/A	N-acetyl cysteine (NAC)	ALP activity	Gel-Ty hydrogels infused with G-NAC (Gel-Ty/G-NAC) exhibited adequate mechanical strength and biocompatibility to encapsulate and facilitate the proliferation of human adipose-derived stem cells (hASCs) across a three-day assessment. AuNPs may facilitate intracellular delivery of NAC. Furthermore, G-NAC facilitated osteodifferentiation both when incorporated into Gel-Ty and when applied directly to hASCs. The osteogenic effects were evidenced by the alkaline phosphatase (ALP) activity assay.	[58]
Imaging agent	40	N/A	poly (L-lysine)	Bright-field microscopy, confocal microscopy and quantification of the incorporation index Viability assay–MTS assay Micro-CT analysis	It is feasible to integrate the AuNP-PLL complex into DPSC and monitor the cellular behavior in a 3D analysis using micro-CT. Additionally, the inclusion of 0.2 mg/mL of AuNP-PLL does not disrupt the fundamental behavior of DPSC.	[59]
	N/A	N/A	None	Optical coherence tomography (OCT)	The addition of AuNPs into root canal irrigants enhances their optical opacity. Gold addition has been found to reduce micro-infiltration at the level of root canal walls, enhancing the adherence of filling materials to dentin.	[60]

#### 3.1.1. General Techniques for AuNPs’ Synthesis and Surface Modification

In 1951, the Turkevich method, one of the most well-known methods for the synthesis of spherical AuNPs, was introduced. It relies on the reduction of HAuCl_4_ by citrate in water [12]. AuNPs can be synthesized using a variety of methods, including physical, chemical and biological processes. One of the most promising approaches is the environmentally friendly “green synthesis”, which has gained significant attention in recent years. The synthesis of AuNPs using plant extracts reduces chemical side effects and avoids creating toxic by-products. Various plant extracts can be used as a reducing agent, whereby Au^3+^ is easily reduced to Au^0^ [41,42,45,50,52,53,61].

Surface modifications of AuNPs play an important role in biomedical applications. Modified molecules such as ligands, proteins, drugs, antibodies and oligonucleotides are decorated on AuNPs’ surface to specifically affect the target cells [16]. This modification cannot only improve the cell adhesion and proliferation capability but also stabilize the diffusion of nanoparticles in biological fluids [8]. Techniques for the surface modification of AuNPs can be divided into two major groups. The first group is physical interactions, including electrostatic and hydrophobic interactions. These interactions are simple and spontaneous processes; however, the interactions are unstable and responsive to various environmental conditions. Due to the electrostatic attraction of negatively charged AuNPs, drugs may bind covalently to AuNPs through amine groups and show better cell penetration [16,62]. On the contrary, positively charged AuNPs showed higher cellular uptake in hMSCs [7]. The second group is chemical interactions, which are more stable and less sensitive to environmental changes, although these interactions required complex processes. The cell targeting can involve both active and passive reactions. AuNPs decorated with specific molecules can bind to specific receptors on the target cell; this is called active targeting. Passive targeting depends on the effectiveness of cellular absorption, which is related to enhanced permeability and retention (EPR) effects [16,63]. In addition, selected molecules coated on AuNPs have a significant impact on targeting specific cells, which increases the mortality and DNA damage of targeted cells and results in fewer systemic side effects [19,64].

#### 3.1.2. Impact of AuNPs’ Size and Shape on the Cellular Uptake of Stem Cells

The size of AuNPs is a fundamental parameter that controls their properties and behaviors. It is a key consideration in the biofunctions and application of AuNPs because the size determines the cellular uptake and biological distribution [15,16,17]. The optimal size, between 25 and 50 nm, is more prone to aggregation in the target cells [18]. However, the rate of cellular uptake is not related to the size of AuNPs [16]. AuNPs adopt a variety of shapes, each with unique properties and applications. The cellular uptake rate is instead related to the shape of AuNPs, which significantly affects the number of AuNPs that accumulate in cells [8,16,46]. The most common shapes are spherical and rod-shaped; these are relatively simple to synthesize, have strong plasmonic properties and allow for precise tuning of the surface plasmon resonance (SPR) wavelength with the highest uptake rate. Although both spherical and rod-shaped AuNPs show a strong cellular uptake rate, spherical AuNPs can promote higher osteogenic induction, osteogenic differentiation, alkaline phosphatase (ALP) activity and calcium deposition of hMSCs than rod-shaped AuNPs [8]. Spherical AuNPs had a stimulatory effect on the proliferation rate and biocompatibility of dental pulp stem cells (DPSCs) derived from exfoliated deciduous teeth (SHED) [65]. Furthermore, cube-shaped AuNPs present a total of 12 edges, whereas nanospheres lack edges. It is plausible that the presence or absence of edges may influence cell adsorption activities [46].

#### 3.1.3. Impact of AuNPs’ Size and Shape on Bacteria Present in the Mouth

The antimicrobial properties of nanoparticles can be attributed to their extremely small size, which can be up to 250 times smaller than that of bacteria. The small size of AuNPs can lead to electrostatic interactions between the Au atom and the negatively charged cell wall of the microbes [45]. Smaller AuNPs also have a higher surface-area-to-volume ratio, leading to stronger SPR effects and the absorption of shorter wavelengths of light. Moreover, smaller AuNPs typically exhibit higher catalytic efficiency, providing more active sites for catalytic reactions [19]. A 25 nm AuNP had more potent antibacterial action than chlorhexidine against evaluated standard species of *S. mutans*, *S. salivarius* and *S. sanguinis* [43]. However, some reports showed that a smaller size of spherical AuNPs (~4 nm) induced a higher cellular uptake and excess ROS than 40 nm AuNPs. In Ibrahim, et al. [66], 5 nm AuNPs showed greater cytotoxicity than 10 and 80 nm AuNPs. In addition, apoptosis and necrosis were activated through ROS generation. The surplus ROS induced higher cytotoxicity and unfavorable adipogenic differentiation rather than osteogenic differentiation [8]. Larger AuNPs, especially 50 nm, can readily penetrate cell membranes and access intracellular compartments, making them suitable for drug delivery and imaging applications [4]. Larger AuNPs, 80 to 100 nm, can adhere on bacterial cells, increasing tension in the membrane and leading to cell rupture [20]. The morphology of AuNPs predominantly comprises spherical and rod-shaped structures, which can be utilized in various applications. AuNPs with alternative geometries, such as star-shaped, have found applications in SERS, photothermal therapy and biological imaging. Furthermore, AuNPs can serve as either core or shell components in core–shell structures. A core–shell structure offers synergistic properties resulting from the combination of different materials, such as enhanced stability, excellent biocompatibility and the ability to be functionalized in drug delivery systems [21].

### 3.2. Application of AuNPs in Dental Biomaterials

**Figure 3:**
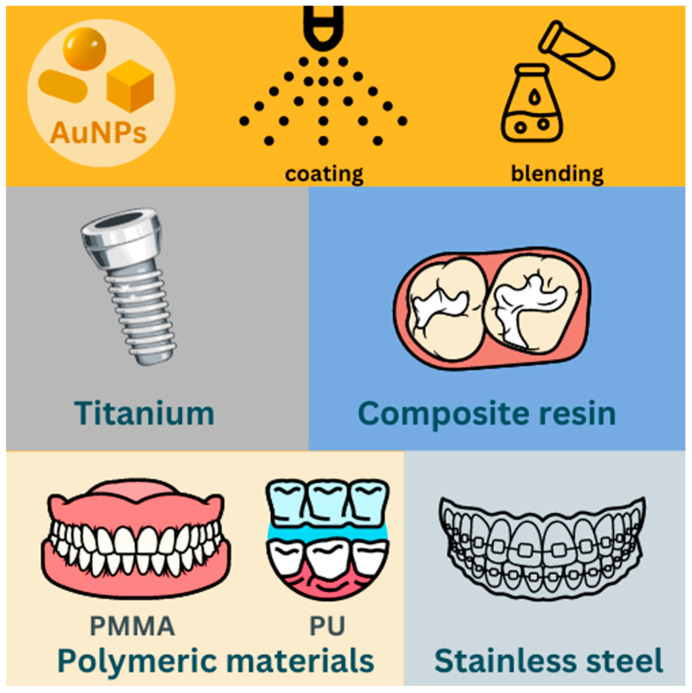
AuNP-modified dental materials.

The incorporation of AuNPs into dental materials has great potential for improving dental treatments and patient outcomes (Figure 3). The dental biomaterial used within the human body should effectively prevent the development of biofilms by pathogens, minimizing the risk of severe consequences [67]. AuNPs exhibit distinctive characteristics, including antibacterial activity, enhancement of mechanical properties and targeted drug delivery capabilities, which make them helpful for enhancing the effectiveness of regenerative and tissue engineering. Moreover, researchers have investigated the application of AuNPs in regulating bacterial pathogenicity in the mouth without causing harm to cells, demonstrating their potential as substitutes for conventional antibiotics in the treatment of periodontal diseases. Coating with AuNPs had an inhibitory effect on the formation, maturation and viability of *S. aureus* biofilms [67]. Pure and modified surfaces of AuNPs with conventional antibiotics exhibit effective antibacterial activity against Gram-negative and Gram-positive bacteria through adhesion and penetration on the cell membrane. Moreover, AuNPs show antiviral activity through interaction with viral surface proteins [19,68,69,70,71,72]. A review of each article about the use of modified AuNPs in dental materials is presented in Table 3.

**Table 3:** AuNPs’ modification of dental materials.

Material	Modification Technique on Materials	Modified Factors of AuNPs			Enhanced Activities	Findings	Reference
		Size (nm)	Shape	Surface Modification			
Titanium	Magnetron sputtering	8–26	Rod	AuPt/PtAu	Osseointegration Antibacterial	AuPt/PtAu TiO_2_ strongly affected the osteogenic performance of the hMSCs when using plasmonic photocatalysis and low-level laser therapy at 470 and 600 nm.	[6]
	Chemisorption: Gold-sulfur bonding (Au-S bond)	30	Spherical	(3-mercaptopropyl) trimethoxysilane (SH(CH_2_)_3_Si(OCH_3_)_3_, 3-MTPMS)	Osseointegration	AuNP-immobilized titanium implant showed higher values in osteogenic differentiation of human bone-marrow-derived MSCs and higher osseointegration parameters than an HA-titanium implant.	[14]
	Chemisorption: Gold-sulfur bonding (Au-S bond) on silanized Ti surface with (3-mercaptopropyl) trimethoxysilane (SH(CH_2_)_3_Si(OCH_3_)_3_, MTPMS)	28	Spherical	None	Osseointegration	Ti-AuNPs significantly enhanced the osteogenic differentiation in human adipose-derived stem cells (ADSCs) and influenced the osseous interface formation.	[22]
	Magnetron sputtering	20–26	Spherical	None	Antibacterial	Au@TNTs exhibit a preferable effect in restricting the growth of *E. coli* and *S. aureus* without cell cytotoxicity.	[73]
	Decomposition of HAuCl_4_ by UV-light illumination	N/A	Spherical	None	Antibacterial and soft tissue healing promotion	An enhanced antibacterial effect against multispecies biofilm was realized via photocatalytic activity triggered by visible-light irradiation and generating a greater quantum yield of reactive oxygen species. AuNP decoration would also exert favorable effects on cell attachment, proliferation and migration fibroblast.	[74]
	Dipping technique (solution immersion)	N/A	N/A	Chitosan, DNA/c-myb	Osseointegration	The results from studying rat mandibles in vivo showed that titanium (Ti) implants coated with Ch-GNP/c-myb increased both the volume and density of newly created bone. Additionally, the inspection using microcomputed tomography revealed improved osseointegration between the dental implant and the bone, particularly in cases of ovariectomized osteoporosis.	[75]
	Decomposition of HAuCl_4_ by UV-light illumination	20	Spherical	None	Antibacterial	AuNPs–TNTs showed excellent biocompatibility and exhibited significantly enhanced antibacterial activity against *P. gingivalis* via ROS generation under a simple ultrasound treatment.	[76]
	Immersion/gel coating with poly(lactic-co-glycolic acid) PGLA solution	15–20	Spherical	AntagomiR204	Osseointegration in type 2 DM (in vivo study)	AuNP-antagomiR204 was released from the PGLA sheet and taken up by adherent BMSCs. This study showed the titanium implant promoting osseointegration in type 2 DM rats.	[77]
	Magnetron sputtering	30–38	Rod	Tetracycline/polycaprolactone (TC/PCL)	Antibacterial activity	AuNPs can produce remote-controlled tetracycline elution using near-infrared laser irradiation.	[78]
	Solution immersion	10	Spherical	None	Osseointegration	Photofunctionalized GNPs can highly improve the osteogenic capabilities with enhanced gene expression of osteogenic markers; Col-1, OPN and OCN; and ALP activity.	[79]
	Solution immersion	N/A	N/A	Chitosan, insulin growth factor binding protein-3 (IGFBP-3)	Osseointegration	Utilizing Ch-AuNPs conjugated with IGFBP-3 as a covering for titanium implants improved the process of bone formation and the integration of dental implants with the surrounding bone tissue against methylglyoxal-induced bone deterioration in a rat model.	[80]
PMMA	Au suspension in PMMA monomer	57–82	Spherical	None	Antibacterial and mechanical properties (microhardness)	PMMA/AuNPs showed a more significant antibiofilm effect against the monomicrobial biofilm formation of *C. albicans*, *S. mitis*, *S. aureus* and *E. coli* than PMMA. AuNPs slightly increased the Vicker’s microhardness of PMMA.	[20]
	Physically combined in a ratio of 0.05 wt/wt % of AuNP powder	10–20	Spherical	None	Mechanical properties affecting dimensional stability	AuNP-modified heat-cured PMMA showed better dimensional stability and decrease in the artificial tooth movement than conventional PMMA.	[23]
	Au suspension in PMMA monomer	11	Spherical	None	Antifungal activity	PMMA/AuNP surfaces were smoother and more hydrophilic with negative charge than pure PMMA surfaces, which reduced the adhesion of *C. albicans.* AuNPs decreased the tensile strength of PMMA significantly.	[27]
	Au suspension in PMMA monomer with concentrations 0.05% and 0.2%	45–65	N/A	None	Mechanical properties affecting flexural strength	The flexural strength of PMMA with the addition of 0.05% AuNPs was significantly greater than with 0.20% AuNPs, whereas differences in sizes of AuNPs added to PMMA did not significantly affect its flexural strength.	[61]
	Au suspension in PMMA monomer	N/A	Spherical	None	Mechanical properties affecting flexural strength and elastic modulus, thermal conductivity, density and hardness	Incorporation of AuNPs into heat-polymerized PMMA resin led to a decrease in the flexural strength and elastic modulus. At the same time, the density, thermal conductivity and hardness increased.	[81]
Polyurethane resins (PU, dental aligner; Invisalign)	Solution immersion	<4 nm of AuDAPTs	N/A	4,6-diamino-2-pyrimidinethiol (DAPT)	Antibacterial	AuDAPT-coated aligners have antibacterial effects on a suspension of *P. gingivalis*, a drug-resistant pathogenic oral bacterium. The strength of these effects depends on the amount of AuDAPT and bacterial concentration. The neighboring area of the material was also affected. AuDAPT-coated aligners slowed biofilm formation and showed favorable biocompatibility.	[82]
Polyacrylic acid (PAA) and polyacrylamide (PAm)	Au suspension in AA or Am monomer	AuCl—57.2 to 69.4 nm AuAc—84.2 to 134.3 nm	AuCl—Spherical AuAc—Ellipsoidal	None	Mechanical properties	Polyacrylate-AuNP composites exhibited lower compressive strength compared to the control samples, while their toughness increased.	[83]
Resin composites	Filler composition	12–15	Spherical	None	Polymerization ratio	AuNPs showed plasmon-enhanced polymerization for green-light-photopolymerizable dental resin with a maximum value at 0.0208 wt%.	[29]
	Filler composition	AuCANPs = 14.7 ± 4.7 nm AuCPNPs = 18.7 ± 9.7 nm	N/A	Citrate (AuCAPNPs) Carboxyphenyl (AuCPNPs)	MMP inhibition and mechanical properties	AuNPs are attractive candidates to inhibit MMPs and improve the mechanical properties of resins without cytotoxic/genotoxic effects on cells, and therefore they should be suitable for applications in adhesive resin systems.	[84]
	Filler composition	5	Spherical	None	Degree of conversion and mechanical property affecting diametral tensile stress	The optimal conditions were a 0.0208 wt% AuNP concentration and 1.4 mW/cm^2^ light intensity, at which the DTS and DC data were all maximal.	[85]
Stainless steels	Solution immersion	3.5–5.5	Nanocore	polyoxoborate matrix	Antibacterial	The use of a nanocomposite consisting of AuNPs incorporated into a polyoxoborate matrix (BOA) had been found to effectively decrease the colonization of orthodontic appliances by *S. mutans*.	[86]

#### 3.2.1. Blending AuNPs in Dental Biomaterials

Polymeric materials are frequently considered potential biomaterials for application in the field of dentistry. Suspensions of AuNPs are incorporated as polymeric monomers or fillers, mainly PMMA and resin composites. PMMA/AuNPs are smoother and more hydrophilic than a pure PMMA surface [27]. Thus, they showed a significant antibiofilm effect in *C. albicans*, *S. mitis*, *S. aureus* and *E. coli* [20]. The presence of PMMA-AuNPs at concentrations above 200 ppm resulted in a considerable reduction (56.67%) in viable fungal cell adhesion in Au^0^. No statistically significant differences were observed between the 200 and 400 ppm loads. The addition of AuNPs may change the way the surface interacts with its surroundings or make it more negatively charged. PMMA-AuNPs exerted a predominantly repulsive effect on the negatively charged fungal cell wall. This procedure was considered essential for the antiadhesion effect [87]. AuNPs are promising candidates for suppressing MMPs by chelating Zn^2+^ bound in the active sites of MMPs and enhancing the mechanical characteristics of resins, without any harmful effects on cells. Consequently, they are well suited for use in adhesive resin systems [84]. AuNPs, as fillers, exhibited plasmon-enhanced polymerization for green light photopolymerization of resin composites [29,85].

#### 3.2.2. Coating AuNPs in Dental Biomaterials

Titanium is the most commonly used material in implant dentistry due to its biocompatibility and mechanical properties. Titanium must be treated to induce polarization of the surface, which can attract osteogenic cells to promote bone regeneration [15,22]. In addition, AuNPs can promote osteoblast differentiation and be strongly immobilized on the titanium surface via Au–S bonding [14,22]. Titanium dioxide nanotubes or titania nanotubes (TNTs) have attractive properties in terms of cytocompatibility, osseointegration and photocatalytic activity. TNTs were prepared from titanium surfaces by electrochemical anodization in a 0.5–1.0 wt % HF solution [73,74,88]. Then, AuNPs were deposited on TNTs’ surface by soaking under UV light illumination [74]. In addition, 3-aminopropyltrimethoxysilane (3-APS) was used as a silane coupling agent for the immobilization of AuNPs on the TNT [88]. Xu et al. found that electrodeposition is an effective method for immobilizing AuNPs on the titanium surface by tuning AuNPs into the titania nanotubes to improve osteogenesis by modulating macrophages’ polarization [15]. Magnetron sputtering and ion plasma sputtering are other methods for coating AuNPs on TNT [73]. Chitosan-AuNPs are conjugated with growth factors and DNA to stimulate bone formation and induce osseointegration of titanium dental implants [75]. Furthermore, AuNPs’ deposition on titania nanotubes causes antibiofilm enhancement and promotes soft tissue healing [74]. The multi-coating of AuPt-NPs on titania nanotubes under visible-light irradiation up to 600 nm plays a crucial role in the antibacterial effect and filopodia behavior of hMSCs, which promoted osteogenic functionality in hMSCs [6]. Moreover, an AuDAPT-coated dental aligner (polyurethane resins) showed not only impressive antibacterial activity against *P. gingivalis* but also favorable prevention of biofilm formation [82]. Utilizing a nanocomposite composed of AuNPs integrated into a polyoxoborate matrix (BOA) has been shown to significantly reduce the colonization of stainless-steel orthodontic appliances by *S. mutans* [86].

#### 3.2.3. Effect of AuNPs on the Mechanical Properties of Dental Biomaterials

AuNPs exhibit promising potential for enhancing the mechanical properties of resins used in adhesive systems with satisfactory biocompatibility. Additionally, these combined properties make AuNPs a valuable component for optimizing adhesive resin systems in various applications. The incorporation of AuNPs into PMMA led to a decrease in tensile strength, flexural strength and elastic modulus [27,81]; however, other mechanical properties, such as density, thermal conductivity and microhardness, increased [20,81]. In contrast, Oyar, Sana, Nasseri and Durkan [61] found that the addition of AuNPs to heat-cured PMMA at lower concentrations (0.05%) significantly increased the flexural strength compared with higher concentrations (0.20%). The compressive strength of the polyacrylate–AuNP composites was greater than that of the control samples, although their toughness was improved [83]. However, the difference in the size of AuNPs did not affect the flexural strength of the PMMA. Even when the highest dose of AuNPs (400 ppm) was added, the PMMA–AuNPs still showed a thermogram comparable to that of the control during the deterioration process. The presence of AuNPs did not affect the glass transition phase of the denture acrylic. Although adding AuNPs to denture acrylic provides an antibacterial benefit, there was a considerable increase in ΔE values as the dose of AuNPs was increased from 100 to 400 ppm [87].

### 3.3. AuNPs and Their Related Host Cells in the Oral Cavity

AuNPs are widely used in the form of coatings on many materials to enhance not only steps in the healing process such as cell adhesion, proliferation and differentiation but also antibacterial, antioxidant and drug-molecule-carrying activities. Gold and its alloys have been used in dental applications and reconstructive surgery. Due to their biocompatibility, durability and inertness, they do not cause an immune response or allergic reactions in the body. AuNPs can conjugate various drug molecules and have specific functions in targeted therapy. The optimal concentration, size and shape of AuNPs for use in nanomaterials are vital factors in stem cell therapies, as well as tissue engineering and regenerative medicine [65,66].

#### 3.3.1. Osteogenic Potential of Stem Cells

The alveolar bone is one of the important hard tissues in the oral cavity that surrounds the teeth. Bone adaptation plays a major role in the healing process of alveolar bone defects that generally result from periodontitis, trauma and infection [89]. Osseointegration plays a major role in successful implant anchorage. Crucial factors that influence the success rates of dental implant placement in low-density bone are implant material, implant design and surgical procedures [90]. Recently, researchers have focused on bone tissue engineering applications utilized by AuNPs [14,22,26]. The mechanism by which AuNPs induce bone repair remains unknown. Prior research has demonstrated that conjugated AuNPs have significant potential for reducing the viability of osteoclasts while also promoting osteogenic development without causing any harm. These nanoparticles possess favorable attributes for the treatment of osteoporotic disorders [30]. A titanium implant surface immobilized by an AuNP layer induced the mRNA expression of osteogenic differentiation-specific biomarkers Runx2, OCN, Col-1, OPN and BMP 2, which resulted in osseous implant interface formation [14,56,57,79,91]. The expression of Runx2 genes in the nucleus is the key osteogenic transcription factor and is upregulated by the *p38/MAPK*, *ERK/MAPK* or *Wnt/β-catenin* signaling pathways, which can be mechanically activated by AuNPs [92]. Moreover, AuNPs regulated the activation of Yes-associated protein (YAP), resulting in osteogenic differentiation [4,56]. AuNPs demonstrated a higher percentage of cell survival in human MG-63 cell lines compared to the control group, indicating their osteoinductive potential [57]. AuNPs may activate cellular autophagy, which affects the cytoskeletal structure to promote osteogenic differentiation [92,93]. In addition, AuNPs inhibit adipogenic differentiation [3,14,22,58,79]. AuNPs promoted the osteogenic functionality of hMSCs under 600 nm visible-light irradiation because of the combined effect of photothermal scattering and visible-light low-level laser therapy (LLLT) [6,94]. Moreover, AuNPs immobilized on the titanium surface led to increased ADSC or MSC proliferation and a maximized level of ALP activity [14,22,79]. The modification of nanostructures affected the viability and differentiation in rat bone marrow MSCs [5]. Xu, He, Zhang, Ma, Zhang and Song [15] illustrated that AuNPs lowered inflammatory responses by reducing microvessel density and mediated the M1–M2 transition of macrophage polarization, which has the potential to promote macrophage-mediated osteogenesis. AuNPs enhanced osteogenic differentiation in a size-dependent manner [4,95]. Smaller AuNPs (25–35 nm) entrapped within the anodized pure titanium surface can promote M2-polarized macrophages and enhance osteogenesis [15]. Furthermore, 30 and 50 nm AuNPs appeared to promote the highest osteogenic differentiation of ADSCs and hMSCs [3,4]. AuNPs of 45 nm could promote osteogenic differentiation of PDL stem cells and bone regeneration more than 13 nm AuNPs through autophagy [93]. Ch-AuNPs/c-myb suppressed osteoclastogenesis and promoted osteogenesis [75]. All sizes and shapes of AuNPs promoted the differentiation of various stem cells toward osteoblasts, but 30–50 nm AuNPs demonstrated an extraordinary capacity for bone regeneration [22]. In many reports, AuNPs of various shapes such as nanospheres, nanostars and nanorods affected osteogenic differentiation [4]. Li, Li, Zhang, Wang, Kawazoe and Chen [4] also found that 40 nm AuNP nanorods inhibited the osteogenesis ability of hMSCs. AuNPs can exhibit not only osteogenic differentiation but also extracellular matrix mineralization [92]. In another study, surface modification did not affect osteogenic differentiation and stimulated osteogenesis by increasing extracellular matrix mineralization. AuNP-COOH showed upregulation of proliferation growth factors of hMSCs (FGF-2 and TGF-β); however, it showed a reduction in ALP activity and matrix mineralization in hMSCs [7].

#### 3.3.2. Gingival Fibroblast and Epithelial Cells

The oral epithelium is the outermost barrier against the oral environment and has contact with vascularized connective tissue. AuNPs can accelerate wound healing by promoting cell migration, angiogenesis and collagen deposition [68]. Moreover, AuNPs/TiO2-NTs also promote fibroblast adhesion, proliferation and migration by upregulating the gene expression levels of fibronectin and pFAK without any irradiation [74]. AuNPs have procoagulant activity, which gives them superior adhesion ability to promote the accumulation of platelets and accelerate the coagulation process. AuNPs can accelerate wound healing due to having denser tissue than new collagen on the CCAu3 group, which illustrates the antioxidant effects and relates to the corresponding gene expression [68]. With the underlying connective tissue, fibroblasts proliferate and migrate into the wound bed and deposit new extracellular matrix, followed by a complex cascade of intracellular biological pathways [68,74]. Thus, the oral epithelium has numerous factors associated with the healing process, for instance, cells, cytokines, saliva and microorganisms. 

#### 3.3.3. Dental Pulp Stem Cells (DPSCs)

The biocompatibility and proliferation rate of DPSCs produced from SHED were shown to be enhanced by spherical AuNPs [65]. The incorporation of a novel calcium phosphate cement made of AuNPs enhanced the performance of human DPSCs. The observed improvement was characterized by an increase in cell adhesion and proliferation, as well as an enhancement in osteogenic differentiation [56]. In situ SERS revealed that mitochondrial nanoprobes (MT-AuNPs/MLS@RGD-PEG-AuNPs) enhanced the accelerated differentiation of DPSCs by increasing the MMP to stimulate more ATP production via the thermoplasmonic effect of AuNPs. The results indicated an increase in the expression of glucose and hydroxyproline within DPSCs during the cell differentiation process [55].

### 3.4. The Possible Use of AuNPs in Clinical Dental Applications

AuNPs have demonstrated significant potential in several clinical dental applications because of their distinctive features, including their compatibility with living organisms, simplicity of modification and optical qualities.

#### 3.4.1. Diagnostic Imaging and Oral Cancer Detection

AuNPs exhibit unique optical properties, including strong absorption and scattering of light due to SPR, which can enhance the contrast in imaging techniques such as X-rays, computed tomography (CT) and magnetic resonance imaging (MRI). Their ability to scatter light and absorb radiation improves the visibility of dental structures and other abnormalities. This phenomenon arises from the collective oscillation of free conduction electrons in the metal when stimulated by incident electromagnetic radiation, which causes the visible color to change [50]. The wavelength at which SPR occurs is determined by several factors, such as the dimensions, morphology and dielectric surroundings of the nanoparticles [63,96]. By controlling these parameters, the SPR band of AuNPs can be specifically tuned across a wide range of wavelengths, from visible to near-infrared (NIR) regions. AuNPs are used as bioimaging applications, as fluorescent probes and X-ray contrast agents at very low concentrations [1,63]. AuNPs have also been employed in biosensors for an early detection of oral cancer. These sensors can detect specific biomarkers associated with cancer, providing a non-invasive and sensitive method for early diagnosis. Functionalized AuNPs can be designed to bind to specific biomarkers or cells, allowing for targeted imaging of particular tissues or pathogens in the oral cavity. In addition, AuNPs can bind many proteins and drugs and can be actively targeted to cancer cells by overexpressing a cell’s biomarkers [97]. Moreover, due to cellular uptake, imaging can localize the intracytoplasmic region of the cell [96]. The AuNP–PLL complex monitored the cellular behavior of DPSCs using micro-CT [59]. 

#### 3.4.2. Therapeutic Applications

AuNPs act as drug carriers conjugated by several biomolecules [62,96,98,99,100]. Functionalization of AuNPs with biomolecules (e.g., antibodies, peptides, DNA) allows for targeted delivery and specific interactions with biological targets. This can be exploited in receptor-mediated tumor cell targeting, enhanced biocompatibility and biorecognition. AuNPs have been used to modify dental materials to impart antibacterial properties that can prevent the formation of biofilms that adhere to surfaces and are often resistant to conventional antibiotics. Researchers have developed materials that can inhibit bacterial growth and reduce the risk of infections by incorporating AuNPs into dental composites or coatings. Therefore, AuNPs are versatile nanocarriers for targeted drug delivery systems in cancer therapy. They have been shown to play crucial roles in cytotoxicity, protection from enzymatic degradation and enhanced cellular uptake [98,101,102]. AuNPs have strong cationic attractions to negatively charged surfaces, so can adhere on the microbial membrane through electrostatic interaction [39,48,103]. Moreover, AuNPs strongly electrostatically adsorbed with lysine, which is the most abundant amino acid on the membrane of Gram-positive bacteria. This caused various distortions in the structure of microbes, such as changes in permeability, osmolarity, electron transport to reach permanent pores and the subsequent loss of cellular content, which causes cell death [12,38,45,69]. On the other hand, some studies found that the thicker peptidoglycan cell walls of Gram-positive bacteria may protect their cells from rupturing. Therefore, AuNPs are more effective against Gram-negative bacteria [20,45]. The strong electrostatic attraction between AuNPs and the bacterial cell wall interrupted adhesin-mediated interaction, which inhibits pathogenic biofilm formation [103]. At greater concentrations, AuNPs were as effective an antibacterial agent against *S. oralis* as chlorhexidine [44]. In addition, a relatively low concentration of AuNPs is able to trigger apoptosis in malignant cells and causes lower cytotoxicity [16,64,96]. AuNPs can easily identify tumor cells or pathogenic microbial cells, due to specific environmental factors such as an acidic pH or hypoxic conditions [18,19,39,98,104]. AuNPs cause a drastic induction of cell cycle arrest and DNA damage [72]. Chitosan–AuNP–curcumin composites had pH-responsive controlled release behaviors that led to significantly improved drug delivery systems under localized acidic conditions [39]. Lysozyme-functionalized gold nanoclusters (AuNCs) can attach the N-acetylglucosamine on the bacterial cell walls through the lysozyme interaction. This binding triggered the detection of bacteria, leading to drug release and subsequent antibacterial activity. [37]. 

A photothermal effect is induced by AuNPs under NIR irradiation, which is commonly used in non-invasive cancer therapy. This can produce heat energy from absorption and scattering light to stimulate cellular apoptosis and denature proteins. This process can be used in photothermal therapy to selectively destroy harmful cells or bacteria in the mouth, such as those associated with oral cancer or periodontitis, when temperatures reach more than 50 °C [19,68,69,105]. However, further research should examine the comparative impact on bacteria in biofilms. However, it is unlikely that the formation of biofilms alone would provide sufficient protection for bacteria against extremely elevated localized temperatures (73 °C) [100]. AuNPs bind various molecules that can be functionalized as targeted therapy by a specific biomarker and act as light-responsive nanomaterials [106]. In addition, AuNPs eliminate biofilms by thermal degradation under NIR light and may be effective alternatives to antibiotics in the treatment of bacterial infections [39,106]. *S. aureus* and *E. coli* can be eliminated by functionalized AuNPs under NIR light, as well as methicillin-resistant *S. aureus* (MRSA) [39,105]. PTT is an effective antibacterial mechanism that does not lead to antibiotic resistance [68]. In summary, an increased concentration of AuNPs results in a greater absorption rate of photon energy from the laser. This energy can then be converted into heat, leading to an elevation in cell temperature. Consequently, the viability of cells, particularly cancer cells, which are sensitive to heat, is reduced [54]. 

AuNPs acting as a photosensitizer absorb photon energy and convert the surrounding oxygen molecules into highly toxic ROS. This is called the photocatalytic process [19,39,74]. Routine PDT is activated by a common photosensitizer (i.e., methylene blue) for eliminating bacterial biofilm. AuNP-conjugated methylene blue was an attractive photosensitizer for efficient ROS generation, including singlet oxygen (^1^O_2_) or superoxide (O_2_^.−^) through resonance energy transfer (RET) from AuNCs to methylene blue. This illustrated similar bacterial growth inhibition and bactericidal effect [107]. AuNP-conjugated methylene blue capped with bovine serum albumin showed significant antimicrobial PDT against *S. mutans* under white-light LED irradiation for approximately 1 min. The AuNC–MB combination did not exhibit any antibacterial activity in the absence of LED light [49]. Conversely, the electrons could be stored in AuNPs and released into the environment without light irradiation. Consequently, the electrons interacted with oxygen to produce ROS, which resulted in the disruption of the bacterial membrane [47,73,74]. Siberian ginseng-AuNPs had increased ROS generation and mitochondrial membrane permeability of B16 cells, leading to the release of proapoptotic proteins [53]. AuNPs directly enter the bacterial cell and can interrupt the ATP synthesis and whole-cell mechanisms, including membrane damage and cellular uptake [38,69]. Conversely, AuNPs exhibit either no antibacterial activity or very weak antibacterial properties [62,108,109,110]. After the Turkevich method, the most common AuNPs preparation method involves the chemical reduction of Au^3+^ salts to Au^0^, which induces ROS formation and can alter macromolecules, resulting in effective antimicrobial activity against a wide variety of microorganisms [38,39,109]. The deposition of gold ions on different cells showed that metal ions can damage DNA, inhibit replication, disturb cellular or membrane proteins and decrease the production of ATP [46]. The concurrent utilization of low-temperature plasma, ROS generation and AuNPs caused significant disruption of cells, resulting in the release of internal components from several cells [48]. LLLT at 470 and 600 nm played an important role in the antibacterial performance of AuPt/PtAu TiO_2_ [6]. Cells irradiated with AuNPs exhibited a notable decrease in MCF-7 cancer cell density in comparison to cells treated with AuNPs and laser separately [54]. Therefore, ROS generation in an aerobic environment can cause the oxidation of protein and nucleic acids, causing cellular damage [38,69,74]. It also led to impairment of mitochondria function, ultimately resulting in cell death [66]. AuNPs decorated on titanium oxide nanotubes showed a photocatalytic memory effect due to their unique heterogeneous structures and were activated not only via NIR and visible-light irradiation according to the size and shape of AuNPs, but also via a simple ultrasound treatment [6,74,111]. ROS formation occurs in an aerobic environment; the anaerobic Gram-positive bacteria *S. mitis* may not be affected by AuNPs [20]. This hypoxic condition occurs when cells do not receive enough oxygen to induce DNA damage caused by free radicals containing oxygen, and it protects the cells from radiation [54].

#### 3.4.3. Restorative Dentistry

AuNPs are being explored for use in dental restorations to improve the mechanical properties and longevity of materials such as PMMA, dental resins and cements. AuNPs enhanced the mechanical characteristics of resins without causing any harmful effects on cells, and therefore should be suitable for applications in adhesive resin systems [84]. Moreover, AuNPs display SPR upon irradiation at specific frequencies, enabling them to improve the polymerization ratio through the plasmonic effect in resin composites and facilitating polymer chain formation around the metallic nanoparticles, which increased the degree of conversion [29,85]. Higher concentrations of AuNPs in PMMA/AuNPs composites resulted in a modest increase in Vicker’s microhardness [20]. The density and thermal conductivity of PMMA were also increased. However, AuNPs decreased the tensile strength, flexural strength and elastic modulus of PMMA [27,81]. The flexural strength values of NPs doped with 400 ppm revealed a substantial increase compared to dosages of 0–200 ppm. The incorporation of NPs might enhance the mechanical strength of denture base structures without adversely affecting the original PMMA material [87]. On the other hand, nanoparticles had the potential to form vast clusters due to concentrations of stress at the sites of agglomeration, which resulted in a considerable attenuation of flexural strength at higher concentrations (0.20%) compared to lower concentrations (0.05%) of the same particle size. Thus, the modification of AuNPs at lower concentrations did not affect the mechanical properties of the materials but enhanced the flexural strength of PMMA. Hence, the addition of AuNPs of varying sizes to PMMA did not have a substantial impact on its flexural strength [61]. Thus, AuNPs can be used for biomedical purposes without decreasing the mechanical properties of the materials.

#### 3.4.4. Regenerative Dentistry

AuNPs can be used to enhance the growth and differentiation of stem cells, potentially aiding in the repair or regeneration of dental tissue (as reviewed in Section 3.3). AuNPs exhibited an interaction with free radicals, resulting in a decrease in DPPH [52]. Spherical AuNPs exhibited the greatest inhibitory activity on the DPPH radical, while polyhedral AuNPs did not show antioxidant properties. AuNPs synthesized from plant extracts also exhibited better antioxidant activity [41,50]. The antioxidant activity of AuNPs was found to be dose-dependent and was shown to be similar to the ability of ascorbic acid (standard) to scavenge DPPH. At a concentration of 50 μg/mL, CuAuNp demonstrated a maximum DPPH scavenging performance of 90.3% [45]. Green AuNPs using the microwave irradiation synthesis method and *Saussurea obvallata* plant extract showed improved antioxidative action at a concentration of 1000 μg/mL [52]. Moreover, heat production from the oscillation of ursodeoxycholic-AuNPs (UDCA-AuNPs) under NIR irradiation can inhibit proinflammatory cytokines [51]. MLS@RGD-PEG-AuNPs accelerated the differentiation of DPSCs through the thermoplasmonic effect of AuNPs by in situ SERS, which effectively regulated mitochondrial metabolism [55]. Green synthesis of red sandal AuNPs inhibited protein denaturation to a degree comparable to commercial painkillers by reducing chemotaxis and the endothelial leukocyte contact [50]. As the concentration of the extract increased, there was a corresponding increase in anti-inflammatory activity that was similar to the conventional medication standard. CuAuNP shown 79.6% greater anti-inflammatory activity at a concentration of 50 μg/mL compared to diclofenac [45].

### 3.5. The Possible Toxicity of AuNPs in the Dental Field

AuNPs demonstrate excellent compatibility with mammalian cells at lower concentrations [42]. The cytotoxicity of AuNPs is correlated with the surface area of the AuNPs because of the size and concentration of particles taken up into the cells. Cellular oxidative stress leads to an elevation in lactate dehydrogenase (LDH) release, the initiation of apoptosis and the formation of intracellular ROS [66]. AuNPs can be regarded as more biocompatible than AgNPs due to the absence of ROS production in their mechanism of action In contrast, ultra-small particles (1–2 nm) tend to be more toxic, not only due to ROS generation but also because their small size can cause irreversible binding to biopolymers [112]. The size of AuNPs affects the distribution of particles to different organs: larger AuNPs are taken up by macrophages and have somewhat restricted distribution while being more widely distributed throughout the systems, particularly the liver and spleen. Particle size and protein adsorption, together with the capacity of certain cells to internalize AuNPs by endocytosis, determine the method of uptake. AuNPs can induce harmful effects on several systems [95]. Previous studies have demonstrated that nanospheres and rods have higher toxicity levels in comparison to star-shaped, flower-shaped and prismatic AuNPs [112]. Moreover, rod-shaped AuNPs caused more toxicity in SHED than spherical-shaped ones [65]. As a result, AuNPs should be carefully chosen in terms of their size, concentration and duration of clinical application [17,65,96].

### 3.6. Future Perspectives on the Use of AuNPs in the Dental Field

AuNPs demonstrate certain essential characteristics resulting from their biological, mechanical and optical properties. Recent developments in dental technology emphasize the use of innovative materials for therapeutic and regenerative purposes. AuNPs offer enormous potential for tissue engineering, specifically in the processes of stem cell proliferation and differentiation, and exhibit excellent biocompatibility. Thus, both normal and compromised conditions could be improved by utilizing AuNPs in dental treatment. Future developments in the dental field involving AuNPs are expected to encompass their use in diagnostic and imaging technologies [63,96], dental implantology [14,22,57,69], pulp tissue regeneration [59] and periodontal regeneration [58,113]. AuNPs have the potential to be used in advanced sensing technologies such as oral cancer detection, oral biomarkers and localizing specifically targeted cells or tissue. Several reviews have highlighted the benefits of modifying AuNPs with titanium implants to improve osseointegration [14,22,57,69]. Furthermore, the favorable biological characteristics of the regeneration of bone and tissues could enhance dental therapy, incorporating pulp regeneration, soft tissue grafting, bone augmentation and soft tissue healing approaches [58,59,113]. The application of AuNPs on implant components, dental appliances, prostheses and aligners has the potential to enhance both the biological response and antibacterial properties. Therefore, the use of AuNPs in dentistry may limit failures and enhance treatment outcomes.

## 4. Conclusions

In conclusion, this review highlights the promising potential of AuNPs in dental materials, particularly through enhancing biological, mechanical and optical properties. Despite the challenges associated with the synthesis and stability of AuNPs, their modifications to titanium, PMMA and resin composites have demonstrated significant benefits, such as improved antibiofilm activity, osteogenesis and mechanical strength. The SPR effect contributes to their application by enhancing resin polymerization. Nevertheless, further research is needed to explore AuNP interactions with other materials, such as zirconia and PEEK, and to develop advanced methods to optimize their use in dental therapies for diverse patient populations. 

## Figures and Tables

**Table 1:** Search strategy for four databases (PubMed, Embase, Scopus, ScienceDirect).

Database	Search Strategy
**PubMed/Medline**	(“dental health services” [MeSH Terms] OR “Dental” [Text Word]) AND (“gold” [MeSH Terms] OR “gold” [Text Word]) AND (“Nanoparticles” [MeSH Terms] OR “Nanoparticles” [Text Word])
**Embase**	(‘dental medicine’ OR ‘dental specialties’ OR ‘dental specialty’ OR ‘dental system’ OR ‘occupational dentistry’ OR ‘pathology, oral’ OR ‘specialties, dental’ OR ‘state dentistry’ OR ‘dentistry’) AND (‘Au nano-particle’ OR ‘Au nanoparticle’ OR ‘gold nano-particle’ OR ‘gold nanoparticles’ OR ‘nano gold’ OR ‘nano-Au’ OR ‘nanogold’ OR ‘nanoparticulate Au’ OR ‘nanoparticulate gold’ OR ‘gold nanoparticle’)
**Scopus**	(“Dental application”) AND (“Gold nanoparticle”)
**ScienceDirect**	(dental applications) AND (gold AND nanoparticle)

## Data Availability

Not applicable.
